# ﻿A new species of *Casearia* Jacq. (Salicaceae) from Central Panama and insights into its phylogenetic position within the genus

**DOI:** 10.3897/phytokeys.236.108651

**Published:** 2023-12-06

**Authors:** Astrid de Mestier, Ernesto Campos Pineda, Marco Cedeño Fonseca, Orlando O. Ortiz

**Affiliations:** 1 Institut für Biologie – Systematische Botanik und Pflanzengeographie, Freie Universität Berlin, Altensteinstraße 6, 14195 Berlin, Germany; 2 Smithsonian Tropical Research Institute, Box 0843-03092, Balboa, Ancón, Panama; 3 Botanischer Garten und Botanisches Museum Berlin, Freie Universität Berlin, Königin-Luise-Str. 6–8, 14195 Berlin, Germany; 4 Herbario Luis Fournier Origgi (USJ), Centro de Investigación en Biodiversidad y Ecología Tropical, Universidad de Costa Rica, Apdo.11501−2060, San José, Costa Rica; 5 Jardin Botánico Lankester, Universidad de Costa Rica, Apdo. 302–7050, Cartago, Costa Rica; 6 Herbario PMA, Universidad de Panamá, Estafeta Universitaria, Panama City, Panama; 7 Coiba Scientific Station (COIBA AIP), Clayton, Ciudad del Saber, Panama

**Keywords:** Molecular phylogeny, Neotropics, new species, Samydeae

## Abstract

We describe here a new species of *Casearia* from Panama based on both morphological and molecular data. *Caseariaisthmica***sp. nov.** is restricted to the mid-elevation cloud forests of Central Panama and presents morphological similarities with two more widespread species, *C.sanchezii* from high elevation areas of El Salvador and Mexico and *C.tremula* from the Caribbean, Central America, and Northern South America. *Caseariaisthmica* differs in presenting pedunculated and congested inflorescences with up to 20 flowers, as well as flowers with 12 stamens and a pubescent style. Phylogenetic analysis based on selected plastid (*petD*, *trnK*-*matK*, *rpl16* and *rps4*-*trnLF*) and nuclear (GBSSI and ITS) markers shows that the new species belongs to subclade B3 of *Casearia*, a lineage that encompasses species from Central America, Mexico and the Caribbean. Results of the morphological and molecular analysis were congruent and allowed a broader understanding of this new taxon, especially regarding its relationships to other *Casearia*.

## ﻿Introduction

*Casearia* Jacq. is a pantropical genus of more than 200 species, therefore being the most species rich genus of the subfamily Samydeae (Salicaceae). In the Neotropics, it is widely distributed and reported from every biome, such as the Amazonian rainforests, the Brazilian Cerrado, the Caribbean and the savannas (Sleumer 1980; Correll and Correll 1982; Liogier 1994; Gutiérrez 2000). Sleumer (1980) provided a taxonomical revision of the whole family Flacourtiaceae (within which *Casearia* was then placed) for the Neotropics, therefore providing insights into its morphological variability. He divided the genus, based on morphological characters, into six sections which were not found to be monophyletic by a recent molecular study (Mestier et al. 2022). Nevertheless, the study showed nine monophyletic subclades. Recently, many new taxa have been described in the Neotropical regions from both South America (Marquete and Mansano 2010, 2013; Alford 2015; Marquete and Torres 2022) and Mesoamerica (Castillo-Campos and Medina Abreo 2003; Linares and Angulo 2005). Here, we describe a new species of *Casearia* from Central America, endemic to Central Panama. *Casearia* species are trees or shrubs that present pellucid dots or lines on the leaves. The limb possesses a pinnate nervation and mostly serrate, crenate, or subentire margins. Flowers are apetalous, with four to five sepals, sometimes more, and mostly uniseriate stamens, alternating with staminodes. *Casearia* usually presents eight to ten stamens, although some species possess a higher number (Warburg 1895; Hutchinson 1967; Sleumer 1980).

Speciation processes are often associated with morphological changes (Losos and Miles 1994; Givnish 2010) and traditionally, species have been described following a morpho-species concept (Stuessy 2009). Nevertheless, providing a phylogenetic framework for a newly described taxon provides valuable insights regarding its relationships with species of the same genus and support for the accuracy of the species delimitation (Padial and De La Riva 2010). Here, we described a new species of *Casearia* endemic to Panama and establish a phylogenetic tree of the genus with the newly described taxa, therefore providing insights into its phylogenetic position within *Casearia*.

## ﻿Materials and methods

### ﻿Taxon sampling and field work documentation

The description of the new species was based on collections that were carried out in Panama between 1992 and 2022 in Panama and Panama Oeste provinces. The documentation of the plant in the field was carried out in April and June 2022, using a Nikon D3100 digital camera with a Nikon DX AF-S Nikkor 18–55 mm lens. Illustrations were made using Adobe Photoshop 2023 software and the PhotoRoom app.

We sampled new sequence data from the proposed new species (two individuals) and from *Caseariasanchezii* J. Linares & Angulo, through a specimen of the Berlin herbarium coming from El Salvador, since the new taxon bears a certain morphological resemblance to this species. Those newly generated sequences were added to the published alignment by Mestier et al. (2023) of the plastid regions: *petD*, *rpl16*, *rps4*-*trnLF* and *trnK*-*matK*, as well as a nuclear data set based on GBSSI and ITS. Fresh silica-dried material and corresponding vouchers of the new species were deposited at B, PMA and SCZ herbaria. Voucher information can be found in Suppl. material 1.

### ﻿Herbarium work and morphological descriptions

Herbarium specimens, including types, were studied from B, PMA, SCZ, and UCH. Also, scanned images from GBIF, JACQ, JSTOR and TROPICOS were examined. In addition to reviewing the types and protologues of other *Casearia* species, the suspected new species were investigated following taxonomic treatments by Sleumer (1980), Pool (2001) and Gonzalez (2007). The descriptions are based on fertile and vegetative material.

### ﻿Conservation status

The conservation status assessment was based on the criteria of the International Union for Conservation of Nature (IUCN Standards and Petitions Committee 2019), using the parameters of number of locations (the number of geographical or ecological areas of occurrence), extent of occurrence (EOO), and/or area of occupancy (AOO). Calculations of EOO and AOO values were computed with GeoCAT (Bachman et al. 2011).

### ﻿Phylogenetic analysis

Wet laboratory procedure followed the protocol of Mestier et al. (2023). We added the newly generated sequences to the alignment by Mestier et al. (2023). A motif-based alignment approach (Löhne and Borsch 2005) in PhyDE v. 0.9971 (Müller et al. 2010) was used (see Suppl. material 1 for the plastid alignment and Suppl. material 2 for the nuclear alignment). Short regions of uncertain homology (hotspots) were excluded, and gaps were coded following the simple indel coding method (Simmons and Ochoterena 2000), as implemented in SeqState v.1.4.1 (Müller 2005) (see Suppl. material 3 for the plastid matrix and Suppl. material 4 for the nuclear matrix).

Bayesian inference was computed using MrBayes v.3.2.7.a (Ronquist et al. 2011) in the high-performance computer cluster “Curta” of the Freie Universität Berlin. Optimal nucleotide substitution models were selected under the Akaike Information Criterion (AIC) with jModelTest v.2.1.7 (Darriba et al. 2012). The summary of character statistics and evolutionary models can be found in Suppl. material 5. Indels were coded with the F81 model, as suggested by Ronquist et al. (2011). We performed four runs each with four chains, sampling every 10,000 generations with 80 million generations for the plastid dataset and 20 million for the nuclear dataset. The average standard deviation of split frequencies and post burn-in effective sampling size (ESS) was used to verify the convergence of the runs. Finally, 10% of the trees were discarded as a burn-in and a 50% majority-rule consensus tree was constructed.

Maximum Likelihood analysis was performed with RAxML v.8.2.12 (Stamatakis 2014) in CIPRES (Miller et al. 2011). The majority-rule consensus tree from 1000 pseudo-replicates with 200 searches was used to estimate rapid bootstrap support (BS). Parsimony analysis was executed using PAUP v.4.0b10 (Swofford 2008) in the high-performance computer cluster “Curta” of the Freie Universität Berlin, using the commands obtained from the parsimony ratchet as implemented in PRAP (Nixon 1999). It allows the inclusion of all characters with equal weight and treat gaps as missing characters. Ratchet settings included 200 iterations, unweighting 25% of the positions randomly and (weight=2) and 100 random additional cycles. Finally, we obtained Jackknife support (JK) values with a single heuristic search within each of 10,000 JK pseudo-replicates, tree bisection-reconnection branch swapping, and 36.79% of characters being deleted in each replicate with PAUP v.4.0b10 (Swofford 2008). We used TreeGraph 2 (Stöver and Müller 2010) to process the trees and added node support values for all inference methods on the Bayesian majority rule topology.

## ﻿Results

### ﻿Phylogenetic analysis

We generated new sequences from plastid and nuclear regions for this study, which we added to the alignment provided by Mestier et al. (2023). The resulting alignment had 7724 positions of which *rps4*-*trnLF* contributed to 2126, *trnK*-*matK* 3137, *petD* 1333 and *rpl16* 1128 (Suppl. material 2). The final matrix had 8017 positions including the indels (Suppl. material 3). Unfortunately, ITS could not be amplified for *C.laetioides* (=*Zuelaniaguidonia* (Sw.) Britton & Millsp), the species resolved as sister in the plastid tree to the putative new species, focus of this investigation. To test if the nuclear genomic partition would support the same relationships, we included information of another nuclear marker, GBSSI. The nuclear alignment (GBSSI and ITS) had 1711 positions (Suppl. material 4) and the final matrix 1647 positions, including the indels (Suppl. material 5).

**Figure 1.**
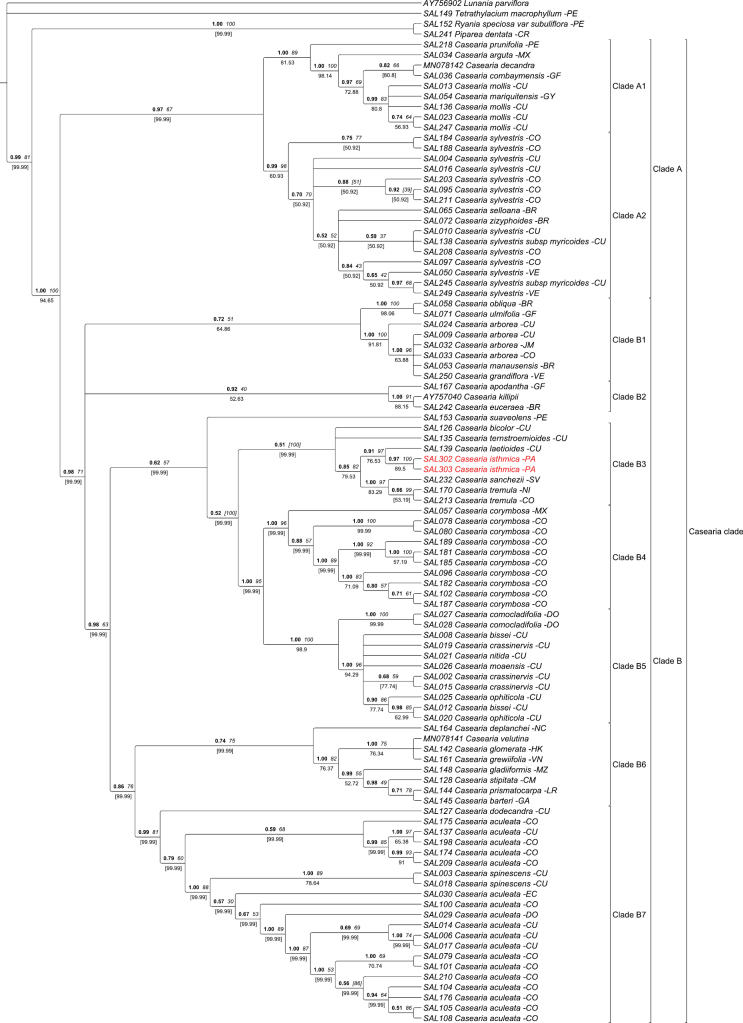
Bayesian 50% majority-rule consensus tree based on four plastid markers (*rps4-trnLF, trnK-matK, rpl16* and *petD*). Values above branches in bold are posterior probabilities (PP), values in italic are bootstrap support (BS) and Jackknife support (JK) is below the branches. Values in square brackets represent a conflict in the topology of the Bayesian analysis with maximum likelihood or Bayesian analysis with parsimony. The species name at the tip of the node is preceded by its DNA number and followed by the country of origin of the sample.

**Figure 2.**
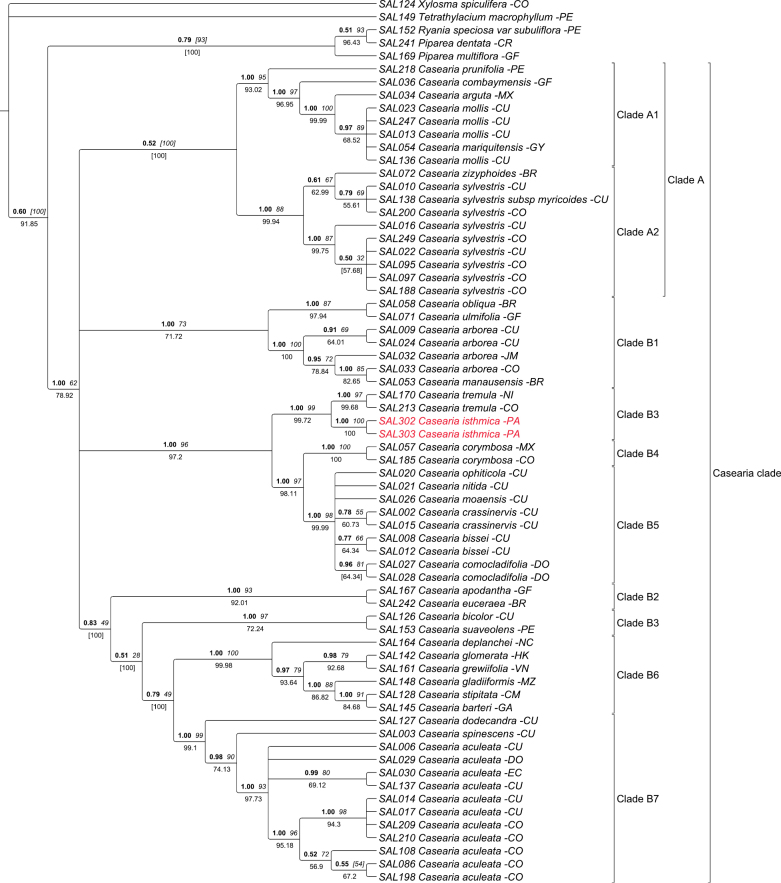
Bayesian 50% majority-rule consensus tree based on two nuclear markers (GBSSI and ITS). Values above branches in bold are posterior probabilities (PP), values in italic are bootstrap support (BS) and Jackknife support (JK) is below the branches. Values in square brackets represent a conflict in the topology of the Bayesian analysis with maximum likelihood or Bayesian analysis with parsimony. The species name at the tip of the node is preceded by its DNA number and followed by the country of origin of the sample.

The plastid and nuclear phylogenies presented in Figs 1 and 2 respectively, are highly congruent and similar to previous molecular studies (Mestier et al. 2022, 2023), recovering the same nine *Casearia* subclades. The newly sequenced individuals of *Caseariaisthmica* are retrieved together in subclade B3 both in the plastid (PP: 0.51. BS: [100], JK: [99,99]) and the nuclear tree (PP: 1, BS: 1, JK: 98.99). In both trees, *C.isthmica* is also retrieved as direct sister to *C.laetioides* (A. Rich.) Northr., to which a lineage of *C.tremula* (Griseb.) Griseb. ex C. Wright and *C.sanchezii* J. Linares & Angulo is sister. In both trees, subclade B4 including the more widespread *C.corymbosa* Kunth and subclade B5 containing the exclusively Caribbean taxa of the genus are resolved as close relative to the subclade B3.

### ﻿Taxonomic treatment

#### ﻿ Casearia isthmica

Taxon classificationPlantaeMalpighialesSalicaceae

de Mestier & O.Ortiz sp. nov.

724A0DAA-225B-5D1F-9EF2-BD8ECFA63B9B

urn:lsid:ipni.org:names:77332275-1

Figs 3 , 4

##### Type.

**Panama. Distrito de Capira**: Parque Nacional Altos de Campana, sendero La Rana Dorada, ca. 100 m desde la entrada, 8°41'32"N, 79°55'34"W, 839 m, 27 April 2022, *E. Campos & J. Sumich 1329* (SAL302) (holotype: PMA-127644!; isotypes: B-101233230!, SCZ! two sheets, barcodes: 20033 and 20034).

*Caseariaisthmica* shares morphological similarities with *C.laetioides* (=*Zuelaniaguidonia* (Sw.) Britton & Millsp), *C.sanchezii* and *C.tremula*. From both the plastid and nuclear trees, *C.laetioides* can be hypothesized as sister species to the newly discovered *C.isthmica*. In terms of morphology, this new taxon differs from *C.laetioides* (A. Rich.) Northr. by having essentially glabrous leaf blades (vs. pubescent), a prominent style (vs. absent or sometimes poorly developed), greenish-white sepals (vs. yellowish), 12 staminodia and stamens (vs. 15–20 staminodia and 20–40 stamens), and glabrous fruits (vs. pubescent). Furthermore, *C.sanchezii* differs morphologically from *C.isthmica* in presenting sessile inflorescence, crenate to subentire leaf blade and a longer petiole of 1 to 2.5 cm. *C.isthmica* differs from *C.tremula* in having congested inflorescences with 12–20 flowers (vs. loose inflorescences with 3 to 10 flowers), 12 stamens arranged in one row (vs. 15 to 24 stamens in two rows), and pubescent styles (vs. glabrous). The discriminating morphological characteristics between *C.isthmica* and its congeners were listed in Table 1. *C.sanchezii* is found in the cloud forest of Mexico and El Salvador at elevation of 1600 to 2000 m, whereas *C.isthmica* is endemic to Central Panama and occurs at 800 m.

**Figure 3.**
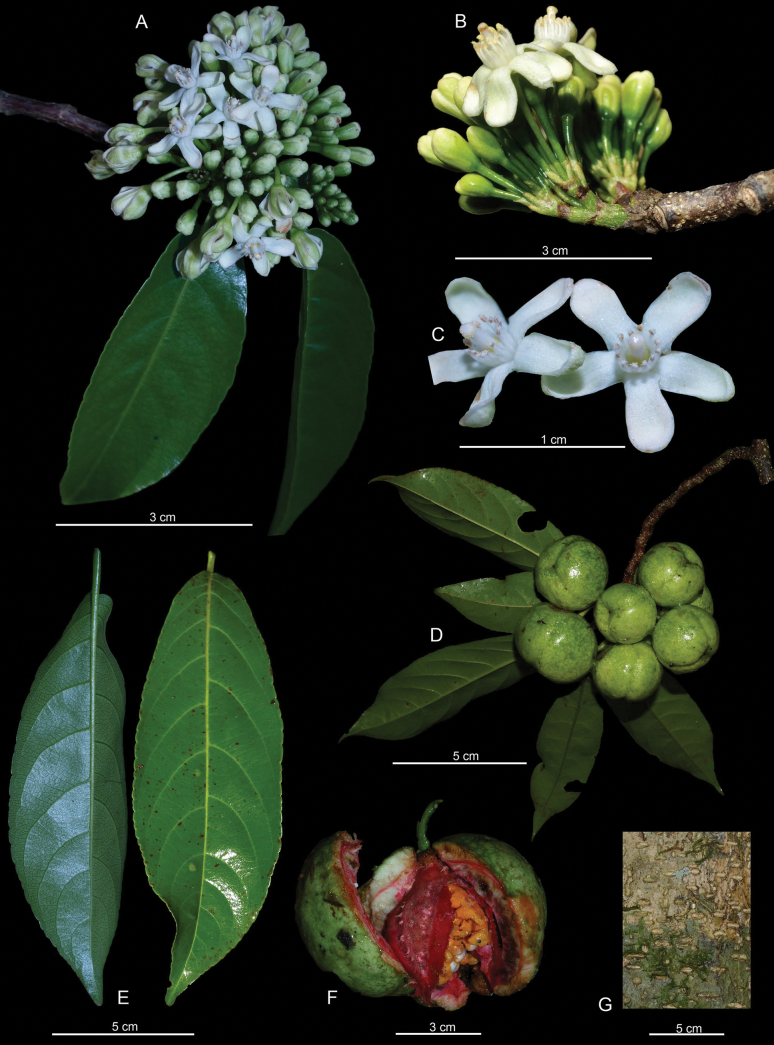
*Caseariaisthmica* de Mestier & O. Ortiz **A** flowering branch **B** inflorescence **C** flowers at anthesis **D** fruiting branch **E** leaves **F** ripe fruit **G** bark. Photos **A, C, E** (back side of the leaf) by Carmen Galdames (Galdames 6153). Photos **D, E** (front side of the leaf) by Carmen Galdames (Galdames 6642). Photo B by Ernesto Campos (Sumich 151). Photos **G, E** by Ernesto Campos Plate by Marco Cedeño-Fonseca.

**Figure 4.**
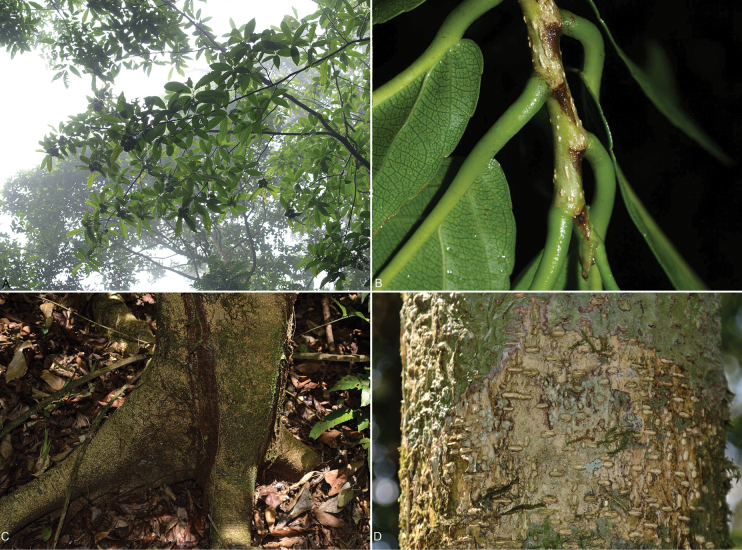
*Caseariaisthmica* de Mestier & O. Ortiz **A** branches **B** twig and stipules **C** trunk base and roots **D** lenticellate bark. Photos **A, B** by Carmen Galdames (Galdames 6642). Photo **C** by Ernesto Campos (Campos 1087). Photo **D** by Ernesto Campos. Plate by Marco Cedeño-Fonseca.

**Table 1.** Comparison of the morphological characteristic of the newly described species and its closest relatives.

	*C.isthmica*	*C.laetioides*	*C.sanchezii*	*C.tremula*
**Elevation (m**)	(600-)800–900	0–500	1600–2200	0–600
**Habitat**	cloud forests	wet or dry lowland forests	cloud forests	wet or dry lowland forests
**Petiole**	caniculate	non-caniculate	caniculate	non-caniculate
**Petiole size (cm)**	0.5–0.8	1–2 cm	(0.4-)1–2.5 cm	(0.5-)1–1.8(-2.4)
**Leaf blade**	glabrous	pubescent	glabrous	glabrous
**Leaf base**	asymmetrical, rounded to acute	asymmetrical, obtuse to rotundate	symmetrical, obtuse, cuneate or subcordate	asymmetrical, cuneate to rounded
**Leaf margins**	serrate to crenate	serrate	crenate to subentire	serrate-crenulate
**Leaf apex**	subcaudate to acuminate	acuminate	acute to acuminate	attenuate to subacuminate
**Inflorescence type**	pedunculate, umbelliform	sessile, fascicle	sessile, umbelliform	pedunculate, fascicle or corymb
**Flower number per inflorescence**	12–20	15	15–20	3–10
**Stamens**	12	20–40	12–15	15–24
**Style**	present, pubescent	absent	present, glabrous	present, glabrous
**Stamens in the same row as staminodia**	yes	yes	yes	no
**Sepals**	5	4–5	5	(5–)6–9
**Sepal color**	greenish-white	yellow	white	greenish-white
**Fruit pubescence**	glabrous	pubescent	glabrous	glabrous
**Fruit color**	red pinkish	yellowish-green	red to dark red	purple red

##### Description.

Tree, up to 15-20 m tall; trunk straight, with bark cream, highly lenticellate; linear lenticels, arranged horizontally and vertically; branches zig-zag in shape, brownish, glabrous, with white lenticels. Stipules 4.0–6.8 × 2.5–4 mm, glabrous, ovate or triangular, persistent on the upper part (distally) and then deciduous. Leaves alternate, simple, deciduous when flowering; petiole 0.5–0.8 cm, canaliculate, glabrous; leaf blade 3.5–8.8 × 0.7–2.6 cm, subcoriaceous or coriaceous, drying brownish or blackish, lanceolate to obovate, asymmetrical, rounded or sometimes acute at the base, subcaudate-acuminate at the apex, densely pellucid punctate, glabrous on both sides, although slightly puberulous on the major abaxial veins; margins slightly serrate to crenate, marginal teeth more frequent in the upper half of the leaf blade; venation pinnate, lateral secondary veins in 5–10 ascending pairs, higher-order veins forming a dense reticulation, prominent on the abaxial surface. Inflorescences pedunculate, congested, umbeliform, each unit 12–20 flowered; peduncle 3 mm; bracts ca. 3.2 mm, coriaceous, ovate-lanceolate, greenish; pedicels ca. 8 mm, terete, basally articulate, green, glabrous. Floral buds oblong-obovate; flowers bisexual; sepals 5, ca. 4.0 × 1.6 mm, seemly free, oblong-obovate, glabrescent, greenish-white, white internally and greenish externally; staminodia 12, white, ligulate, white-hirsute; stamens 12 on the same row as the smaller staminodia and alternating with them, densely pubescent; filaments equal, free, white-hirsute; anthers elliptic, creamy, white-hirsute externally; ovary greenish, ovate, white-hirsute; style undivided, white-hirsute; stigma capitate, creamy. Fruits fleshy, orbicular, up to 5 cm diameter, very glossy, green during development, turning black-purple externally and pinkish or red internally when ripe, covered on the lower part by the sepals, dehiscent by 3 valves; seeds many, creamy-white, aril orange.

##### Distribution and habitat.

**Figure 5.**
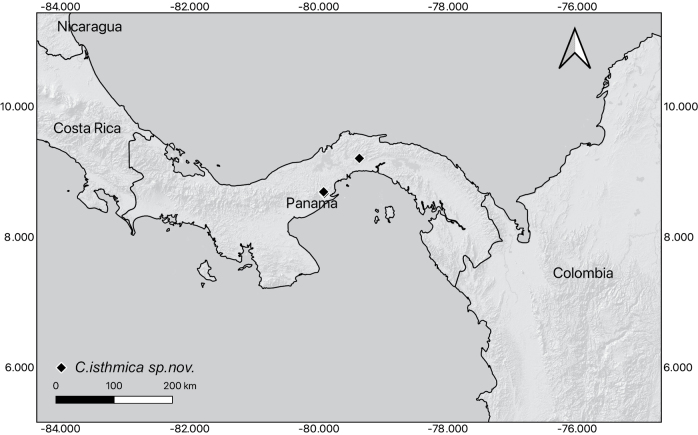
Geographic distribution of *Caseariaisthmica*.

Endemic to Central Panama (Panamá and Panamá Oeste provinces) (Fig. 5), documented only from cloud forests at elevations between (600–)800–900 m, in the premontane rain forest life zone.

##### Phenology.

Flowering in April, May. Fruiting in June, July.

##### Etymology.

The specific epithet refers to the geographic distribution of the new species, which is restricted to the Isthmus of Panama.

##### Preliminary conservation status.

*Caseariaisthmica* is known from ten collections made in two locations (Altos de Campana and Altos de Pacora). These collections were made within or very close to the external limits of protected areas (Altos de Campana National Park and Chagres National Park). Although they are protected areas, both locations face moderate anthropic disturbances, mainly in the borders such as Altos de Pacora (Chagres National Park). The highest threat facing this taxon is the loss of habitat caused by new road constructions and building of housing, as well as destructive tourist activities that are not environmentally sustainable, such as clandestine motorcycle races through the forest. Because this species has a limited distribution (EOO: 220 km^2^; AOO: 16 km^2^), the effect on its natural habitat may be extremely critical and may compromise its conservation. *Caseariaisthmica* must be considered as Endangered [EN B1ab(iii)+2ab(iii)].

##### Selected specimens examined.

**Panama Panamá Oeste Province**: Parque Nacional Altos de Campana; Sendero de Interpretación; 1 km del campamento de los guardaparques de INRENARE; bosque muy húmedo tropical premontano; Camino Zamora; 8°40'N, 79°55'W; 800–900 m; fl; 21 May 1992; *M.D. Correa et al. 8944* (PMA) • Parque Nacional Altos de Campana; colectado a 5 m de la orilla de la carretera dentro del parque; 8°40'N, 79°55'W; 800–900 m; fr; 24 June 1993; *M.D. Correa & E. Montenegro 9622* (PMA) • Parque Nacional Altos de Campana; Finca García; 8°40'N, 79°55'W; 800–900 m; fr; 28 July 1994; *M.D. Correa & E. Montenegro 10717* (PMA) • Parque Nacional Altos de Campana; 3 agosto 1995; *E. Montenegro 1112* (SCZ) • Parque Nacional Altos de Campana; Las Nubes; División Continental entre Finca de Tomás Herrera; 600–700 m; 3 May 1997; *M.D. Correa et al. 11430* (F; MO PMA) • Parque Nacional Altos de Campana; Sendero El Tigre; bosque nuboso; 8°40'N, 79°55'W; 900 m; fr; 11 Jul 1998; *C. Galdames*; *E. Montenegro & H. Valdéz 4317* (F; PMA; SCZ) • Distrito de Capira; Parque Nacional Altos de Campana; colectado en el sendero de la Rana Dorada; ca. A 100 m de la entrada; 8°41'32"N, 79°55'34"W; 839 m; fl; 3 May 2018; *E. Campos & C. Galdames 1087* (B; SCZ) • Distrito de Capira; Parque Nacional Altos de Campana; sendero La Rana Dorada; ca. 100 m desde la entrada; 8°41'32"N, 79°55'34"W; 839 m; fl; 27 April 2022; *J. Sumich & E. Campos 151* (SCZ) • Distrito de Capira; Parque Nacional Altos de Campana; 27 April 2022; sendero La Rana Dorada; *J. Sumich 127* (SCZ). **Panamá Province**: Cerro Pelón; finca del Sr. Rodrigo Coba; reserva boscosa privada; adyacente a Cerro Jefe; 9°12'29"N, 79°22'33"W; 825 m; fr; 29 June 2010; *C. Galdames*; *R. Vergara & F. Rodríguez 6642* (PMA; SCZ).

## ﻿Discussion

Results of both the molecular and morphological analysis highly support the recognition of *Caseariaisthmica* as a new species. Based on the molecular trees (Figs 1, 2), *C.isthmica* can be hypothesized as sister to *C.laetioides* (A. Rich.) Northr. (=*Zuelaniaguidonia* (Sw.) Britton & Millsp) and both are related to taxa with a largely central American distribution such as *C.tremula* (Griseb.) Griseb. ex C. Wright, and *C.sanchezii* J. Linares & Angulo.

Morphologically, *Casearialaetioides*, *C.tremula*, and *C.sanchezii* share similarities with *C.isthmica*, such as the general shape of the leaves, the presence of more than ten stamens with pubescent filaments, and the size of the fruit. However, *C.laetioides* differs from *C.isthmica* in many aspects, mainly in the petiole, leaf blades, inflorescences, fruits, and other floral aspects related to the style and number of stamens and staminodia (Table 1). Character states such as essentially glabrous leaves (sometimes puberulent along the major veins), glabrous and glossy fruits shared between *C.isthmica*, *C.tremula* and *C.sanchezii* (Table 1) could be of plesiomorphic nature but the latter two differ mainly in the type of inflorescence, number of flowers per inflorescence and the number of stamens. Taken together, morphological differences support the recognition of *C.isthmica* as a new species.

*Casearialaetioides* was historically classified within the genus *Zuelania* A. Rich., which has recently been shown to be nested within *Casearia*, on the basis of morphological and molecular characters (Samarakoon and Alford 2019; Mestier et al. 2022). *C.laetioides* is widely distributed in the Caribbean region, in Mexico, in Central America and Northern South America (Sleumer 1980). *Zuelania* was distinguished from *Casearia* by having a high number of stamens and a sessile and peltate stigma, whereas most *Casearia* species possess up to 12 stamens and a capitate stigma with a style. However, it seems that the high number of stamens is a homoplastic character state, as within clade B3, *C.isthmica* is the only species presenting 12 stamens.

From a geographical and ecological viewpoint, *Caseariaisthmica* and *C.laetioides* are both present in Panama, although not in the same ecosystem as *C.isthmica*, which occurs at higher elevation in cloud forest, whereas *C.laetioides* occurs at lower elevation, below 600 m, usually in wet or dry habitats such as seasonal forests (Table 1). Further research will be needed to clarify if *C.laetioides* is monophyletic and a vicariant to *Caseariaisthmica* and to clarify the position of the lineage including *C.isthmica* and *C.laetioides* within subclade B3 of *Casearia*. It will be relevant to a better understanding of the speciation history of *C.isthmica* as an example for the origin of cloud forest species in Panama, but also with respect to the origin of the flora of the Caribbean, for which studies in recent years underscored the close affinities between Central America, Mexico and the Caribbean islands (Cervantes et al. 2016; Mestier et al. 2022).

Some specimens from Panama previously identified as *C.tremula* were re-identified and included in this work as *Caseariaisthmica* (*Galdames et al. 4317* and *Correa et al. 11430*). However, there is currently a representative specimen (*Carrasquilla 2072* MO, PMA) that confirms the occurrence of *C.tremula* in the country, which was collected in the dry seasonal lowland forests from the Pacific slope of Panama.

## Supplementary Material

XML Treatment for Casearia isthmica

## ﻿Additional information

### Author ORCIDs

Astrid de Mestier https://orcid.org/0000-0002-9976-8196

Ernesto Campos Pineda https://orcid.org/0000-0001-8606-5785

Marco Cedeño Fonseca https://orcid.org/0000-0003-0119-0427

Orlando O. Ortiz https://orcid.org/0000-0002-7805-00460

### Data availability

All of the data that support the findings of this study are available in the main text or Supplementary Information.

## Supplementary materials

Supplementary material 1 Taxa used for molecular analysis Data type docx Explanation note Accessions are listed in alphabetic order. Name and author, lab number, DNA bank number, locality, collector and collector number, herbarium voucher and NCBI accession number for *rps4*-*trnLF*, *trnK*-*matK*, *rpl16*, *petD*, GBSSI and ITS respectively. Species names follow Sleumer (1980). ^1^Korotkova et al. (2009), ^2^Li et al. (2019), ^3^Alford (2005), all others are newly sequenced for this study; “–” indicates missing sequences. https://binary.pensoft.net/file/948455This dataset is made available under the Open Database License (http://opendatacommons.org/licenses/odbl/1.0/). The Open Database License (ODbL) is a license agreement intended to allow users to freely share, modify, and use this Dataset while maintaining this same freedom for others, provided that the original source and author(s) are credited.Astrid de Mestier, Ernesto Campos Pineda, Marco Cedeño Fonseca, Orlando O. Ortiz

Supplementary material 2 Annotated alignment of five concatenated plastid regions (*rps4-trnL-F*, *trnK-matK*, *rpl16*, *petD*) Data type fas https://binary.pensoft.net/file/948456This dataset is made available under the Open Database License (http://opendatacommons.org/licenses/odbl/1.0/). The Open Database License (ODbL) is a license agreement intended to allow users to freely share, modify, and use this Dataset while maintaining this same freedom for others, provided that the original source and author(s) are credited.Astrid de Mestier, Ernesto Campos Pineda, Marco Cedeño Fonseca, Orlando O. Ortiz

Supplementary material 3 Matrix of five concatenated plastid regions (*rps4-trnL-F*, *trnK-matK*, *rpl16*, *petD*) including the indel partition Data type fas https://binary.pensoft.net/file/948457This dataset is made available under the Open Database License (http://opendatacommons.org/licenses/odbl/1.0/). The Open Database License (ODbL) is a license agreement intended to allow users to freely share, modify, and use this Dataset while maintaining this same freedom for others, provided that the original source and author(s) are credited.Astrid de Mestier, Ernesto Campos Pineda, Marco Cedeño Fonseca, Orlando O. Ortiz

Supplementary material 4 Annotated alignment of nuclear region (GBSSI and ITS) Data type fas https://binary.pensoft.net/file/948458This dataset is made available under the Open Database License (http://opendatacommons.org/licenses/odbl/1.0/). The Open Database License (ODbL) is a license agreement intended to allow users to freely share, modify, and use this Dataset while maintaining this same freedom for others, provided that the original source and author(s) are credited.Astrid de Mestier, Ernesto Campos Pineda, Marco Cedeño Fonseca, Orlando O. Ortiz

Supplementary material 5 Matrix of nuclear regions (GBSSI and ITS) including the indel partition Data type fas https://binary.pensoft.net/file/948459This dataset is made available under the Open Database License (http://opendatacommons.org/licenses/odbl/1.0/). The Open Database License (ODbL) is a license agreement intended to allow users to freely share, modify, and use this Dataset while maintaining this same freedom for others, provided that the original source and author(s) are credited.Astrid de Mestier, Ernesto Campos Pineda, Marco Cedeño Fonseca, Orlando O. Ortiz

Supplementary material 6 Summary of characters and tree statistics and evolutionary models for each dataset Data type xlsx https://binary.pensoft.net/file/948460This dataset is made available under the Open Database License (http://opendatacommons.org/licenses/odbl/1.0/). The Open Database License (ODbL) is a license agreement intended to allow users to freely share, modify, and use this Dataset while maintaining this same freedom for others, provided that the original source and author(s) are credited.Astrid de Mestier, Ernesto Campos Pineda, Marco Cedeño Fonseca, Orlando O. Ortiz
