# Supplementary material for: ﻿A new species of Casearia Jacq. (Salicaceae) from Central Panama and insights into its phylogenetic position within the genus
Source: PhytoKeys. 2023 Dec 6;236:97–112. doi: 10.3897/phytokeys.236.108651 (PMC10719937; doi:10.3897/phytokeys.236.108651)
Supplement: Supplementary material 1 — Taxa used for molecular analysis [file phytokeys-236-097_article-108651__-s001.docx]

**Appendix** Taxa used for molecular data (Name and author, Labnumber, DNA bank number, locality, collector and collector number, herbarium voucher and NCBI accession number for rps4/trnLF, trnK/matK, rpl16, petD and ITS respectively). Herbaria acronyms for voucher locations follow Index Herbariorum (Thiers, B. [continuously updated]. Index Herbariorum: A global directory of public herbaria and associated staff. New York Botanical Garden's Virtual Herbarium. http://sweetgum.nybg.org/science/ih/). Species names are used following Sleumer (1980). Accessions are listed in alphabetic order. Abbreviations: s.n. = sine numero (without number). ^1^Li & al. (2019), all other are newly sequenced for this study, “-“ indicates missing sequences

**Outgroups: *Lunania parviflora*** Spruce ex Benth., *Alford & Grandez 3114* (BH), AY756902; ***Piparea dentata*** Aubl., SAL241, Costa Rica, *Salazar 2638* (BH), OU452357, OU452365, OU452355, OU452353; ***Ryania speciosa var. subuliflora*** (Sand.) Monach., SAL152, DB 44793, Peru, *M. Rimachi Y. 8081* (BR), HG998004, HG997922, HG998104, FR990829; ***Tetratylacium macrophyllum*** Poepp., SAL149, DB 44790, Peru: Loreto, *M. Rimachi Y. 8224* (BR), HG997921, HG998103, FR990828;

**Ingroups: *Casearia aculeata*** Jacq., SAL006, DB 44708, Cuba: Guantanamo, *T. Borsch 4284* (HAJB, B), HG997945, HG997867, FR990780; ***Casearia aculeata*** Jacq., SAL014, DB 44716, Cuba: Villa Clara, *T. Borsch 5243,* (HAJB, B), HG997951, HG997871, HG998057, FR990786; ***Casearia aculeata*** Jacq., SAL017, DB 44719, Cuba: Holguin, *S. Fuentes et al. 442* (HAJB, B), HG997954, HG997874, HG998060, FR990789; ***Casearia aculeata*** Jacq., SAL029, DB 27861, Dominica: Pedernales, *W. Greuter 26587* (B), HG997964, HG997884, HG998070, FR990798; ***Casearia aculeata*** Jacq., SAL030, DB 27862, Ecuador: Guayas, *E. Asplund 15213* (B), HG997965, HG997885, HG998071, FR990799; ***Casearia aculeata*** Jacq., SAL079, Colombia: Bolivar, *M.C. Martinez-Habibe et al. 2510* (UNO), HG997978, HG997898, HG998082; ***Casearia aculeata*** Jacq., SAL100, Colombia: La Guajira, *M.C. Martinez-Habibe et al. 2591* (UNO), HG997985, HG997905, HG998088; ***Casearia aculeata*** Jacq., SAL101, Colombia: La Guajira, *M.C. Martinez-Habibe et al. 2593* (UNO), HG997986, HG997906, HG998089; ***Casearia aculeata*** Jacq., SAL104 , Colombia: Bolivar , *M.C. Martinez-Habibe et al. 2605* (UNO), HG997988, HG997908, HG998091; ***Casearia aculeata*** Jacq., SAL105, Colombia: Bolivar, *M.C. Martinez-Habibe et al. 2606* (UNO), HG997989, HG997909, HG998092; ***Casearia aculeata*** Jacq., SAL108, Colombia: Bolivar, *M.C. Martinez-Habibe et al. 2611* (UNO), HG997990, HG997910, HG998093, FR990816; ***Casearia aculeata*** Jacq., SAL137, DB 44778, Cuba: Artemisa, *S. Fuentes et al. 1977* (HAJB, B), HG997997, HG997916, HG998098, FR990823; ***Casearia aculeata*** Jacq., SAL174, Colombia: Bolivar, *M.C. Martinez-Habibe et al. 2929* (UNO), HG998009, HG998109; ***Casearia aculeata*** Jacq., SAL175, Colombia: Bolivar, *M.C. Martinez-Habibe et al. 2938* (UNO), HG998010, HG997928, HG998110; ***Casearia aculeata*** Jacq., SAL176, Colombia: Bolivar, *M.C. Martinez-Habibe et al. 2940* (UNO), HG998011, HG998111; ***Casearia aculeata*** Jacq., SAL198, Colombia: Bolivar, *M.C. Martinez-Habibe et al. 3000* (UNO), HG998019, HG997935, HG998119, FR990838; ***Casearia aculeata*** Jacq., SAL209, Colombia: La Guajira, *M.C. Martinez-Habibe et al. 2745* (UNO), HG998021, HG997937, HG998121, FR990840; ***Casearia aculeata*** Jacq., SAL210, Colombia: La Guajira, *M.C. Martinez-Habibe et al. 2756* (UNO), HG998022, HG997938, HG998122, FR990841; ***Casearia apodantha*** (Kuhlm.) de Mestier, Celis & Borsch, SAL167, DB 44802, French Guyana, *O. Lachenaud 2143* (P), HG998007, HG997926, HG998107, FR990833; ***Casearia arborea*** (Rich.) Urb., SAL009, DB 44711, Cuba: Guantanamo, *T. Borsch et al. 4845* (HAJB, B), HG997947, HG998055, FR990782; ***Casearia arborea*** (Rich.) Urb., SAL024, DB 44726, Cuba: Holguin, *S. Fuentes et al. 1370* (HAJB, B), HG997959, HG997880, HG998065, FR990794; ***Casearia arborea*** (Rich.) Urb., SAL032, DB 27864, Jamaica: Parish of Clarendon, *E. Köhler 231* (HAJB, B), HG997966, HG997886, HG998072, FR990800; ***Casearia arborea*** (Rich.) Urb., SAL033, DB27865, Colombia: Antioquia, *R. Fonnegra 6711* (B), HG997967, FR990801; ***Casearia arguta*** Kunth, SAL034, DB27866, Mexico: Chiapas, *E. Martinez M-26281* (B), HG997968, HG997887, HG998073, FR990802; ***Casearia barteri*** Mast., SAL145, DB 44786, Gabon: Moyen-Ogooué, *F. J. Breteler 13106* (BR), HG998001, HG997919, HG998102, FR990826; ***Casearia bicolor*** Urb., SAL126, DB33090, Guyana: Rupununi, *M. J. Jansen-Jacobs 2495* (B), HG997992, HG997911, FR990818; ***Casearia bissei*** J.E. Gut., SAL008, DB 44710, Cuba: Guantanamo, *M. Ackerman 862* (HAJB, B), HG997946, HG997868, HG998054, FR990781***; Casearia bissei*** J.E. Gut., SAL012, DB 44714, Cuba: Guantanamo, *T. Borsch et al. 4428* (HAJB, B), HG997949, HG997869, HG998056, FR990784; ***Casearia combaymensis*** Tul., SAL036, DB27868, French Guyana, *J.J. de Granville 13157* (B), HG997969, HG997888, HG998074, FR990803; ***Casearia comocladifolia*** Vent., SAL027, DB 44729, Cuba: Independancia, *S. Fuentes et al. 1164* (HAJB, B), HG997962, HG997882, HG998068, FR990796; ***Casearia comocladifolia*** Vent., SAL028, DB 44730, Cuba: Barahona, *S. Fuentes et al. 1212* (HAJB, B), HG997963, HG997883, HG998069, FR990797*;* ***Casearia corymbosa*** Kunth, SAL057, DB27889, Mexico: Oaxaca, *Misael Elorsa C. 478* (B), HG997973, HG997892, HG998077, FR990805; ***Casearia corymbosa*** Kunth, SAL078, Colombia: Bolivar, *M.C. Martinez-Habibe et al. 2502* (UNO), HG997897, HG998081; ***Casearia corymbosa*** Kunth, SAL080, Colombia: Bolivar,  *M.C. Martinez-Habibe et al. 2512* (UNO), HG997979, HG997899, HG998083; ***Casearia corymbosa*** Kunth, SAL096, Colombia: La Guajira, *M.C. Martinez-Habibe et al. 2572* (UNO), HG997983, HG997903, HG998086; ***Casearia corymbosa*** Kunth, SAL102, Colombia: La Guajira, *M.C. Martinez-Habibe et al. 2598* (UNO), HG997987, HG997907, HG998090; ***Casearia corymbosa*** Kunth, SAL181, Colombia: Magdalena, *M.C. Martinez-Habibe et al. 2973* (UNO), HG998012, HG997929, HG998112; ***Casearia corymbosa*** Kunth, SAL182, Colombia: Magdalena, *M.C. Martinez-Habibe et al. 2976* (UNO), HG998013, HG997930, HG998113; ***Casearia corymbosa*** Sw., SAL185, Colombia: Magdalena, *M.C. Martinez-Habibe et al. 2982* (UNO), HG998015, HG997932, HG998115, FR990836; ***Casearia corymbosa*** Kunth, SAL187, Colombia: Magdalena, *M.C. Martinez-Habibe et al. 2986* (UNO), HG998016, HG997933, HG998116; ***Casearia corymbosa*** Kunth, SAL189, Colombia: Magdalena, *M.C. Martinez-Habibe et al. 2988* (UNO), HG998018, HG997934, HG998118; ***Casearia crassinervis*** Urb., SAL002, DB 44704, Cuba: Holguin, *T. Borsch et al.* 4056 (HAJB, B), HG997942, HG998052, FR990778; ***Casearia crassinervis*** Urb., SAL015, DB 44717, Cuba: Holguin, *N. Köster et al. 2724* (HAJB, B), HG997952, HG997872, HG998058, FR990787; ***Casearia crassinervis*** Urb., SAL019, DB 44721, Cuba: Holguin, *S. Fuentes et al. 453* (HAJB, B), HG997956, HG997876, HG998062; ***Casearia decandra*** Jacq., MN078142^1^ (complete genome); ***Casearia deplanchei*** Sleumer, SAL164, DB 44875, New Caledonia, *G. Dagostini 783*, P 04819071, HG998006, HG997925, HG998106, FR990832; ***Casearia dodecandra*** (Jacq.) T. Samar. & M.H. Alford, SAL127, DB 33091, Dominica: Pedernales, *W. Greuter 26585* (B), HG997993, HG997912, HG998095, FR990819; ***Casearia euceraea*** de Mestier, Celis & Borsch, SAL242, Brazil, *G. Hobbes 213* (BH), OU452358, OU452366, OU452356, OU452354; ***Casearia gladiiformis*** Mast., SAL148, DB 44789, Mozambique, *P. C. M. Jansen 7670*, BR 0000015960818, HG998003, HG997920, FR990827; ***Casearia glomerata*** Roxb., SAL142, DB 44783, Hong Kong, *L. Tsuen 12687* (BR), HG998000, HG997918, HG998101, FR990825; ***Casearia grandiflora*** Cambess., SAL250, *F. Michelangeli & Mac Alford 692* (BH); ***Casearia grewiifolia*** Vent., SAL161, DB 44874, Vietnam, *U. Swenson 1541* (P), HG997924, HG998105, FR990831; ***Casearia isthmica.***, SAL302, Panama, *Campos 1329* (SCZ, B); ***Casearia isthmica.***, SAL303, Panama, *Sumich 127* (B); ***Casearia killipii*** (Monach.) de Mestier, Celis & Borsch, *Alford & Grandez 3119*, AY757040^3^*;* ***Casearia laetioides*** (A. Rich.) Northr., SAL139, DB 44780, Cuba: Pinar del Rio, *T. Borsch et al. 5954* (B), HG997999, HG998100; ***Casearia manausensis*** Sleumer, SAL053, DB27885, Brasil: Amazonas, *J. L. Santos 855* (B), HG997971, HG997890, HG998076; ***Casearia mariquitensis*** Kunth, SAL054, DB27886, Guyana: Rupununi, *M. J. Jansen-Jacobs 4348* (B), HG997972, HG997891, FR990804; ***Casearia moaensis*** Vict., SAL026, DB 44728, Cuba: Holguin, *S. Fuentes et al. 1689* (HAJB, B), HG997961, HG998067, FR990795; ***Casearia mollis*** Kunth, SAL013, DB 44715, Cuba: Villa Clara, *T. Borsch et al. 5138* (HAJB, B), HG997950, HG997870, FR990785; ***Casearia mollis*** Kunth, SAL023, DB 44725, Cuba: Matanzas, *T. Borsch et al. 5611* (HAJB, B), HG997879, HG998064, FR990793; ***Casearia mollis*** Kunth, SAL136, DB 44777, Cuba: Artemisia, *S. Fuentes et al.1976* (HAJB, B), HG997996, HG997915, HG998097, FR990822; ***Casearia mollis*** Kunth, SAL247, Cuba, *T. Borsch et al. 5926* (HAJB, B); ***Casearia nitida*** Jacq., SAL021, DB 44723, Cuba: Las Tunas, *S. Fuentes et al.* *841* (HAJB, B), HG997958, HG997878, FR990791; ***Casearia obliqua*** Sprengel , SAL058, DB27890, Brasil: Parana, *R. Kummrow 3000* (B), HG997974, HG997893, FR990806; ***Casearia ophiticola*** Vict., SAL020, DB 44722, Cuba: Holguin, *S. Fuentes et al. 656* (HAJB, B), HG997957, HG997877, HG998063, FR990790; ***Casearia ophiticola*** Vict., SAL025, DB 44727, Cuba: Holguin, *S. Fuentes et al. 1645* (HAJB, B), HG997960, HG997881, HG998066; ***Casearia prismatocarpa*** Mast., SAL144, Liberia, *C. C. H. Jongkind 9804* (BR); ***Casearia prunifolia*** Kunth, SAL218, DB 44851, Peru: Loreto, *A. Gentry 29158* (JBGP), HG998025, HG997941, FR990843; ***Casearia sanchezii***, SAL232, El Salvador, *Carballo, R.A. 336* (B); ***Casearia selloana*** Eichler, SAL065, DB27897, Brasil: Paraiba do Sul, *Schwacke 3215* (B), HG997975, HG997894, HG998078; ***Casearia spinescens*** (Sw.) Griseb., SAL003, DB 44705, Cuba: Holguin, *T. Borsch et al. 4115* (HAJB, B), HG997943, HG997866, HG998053, FR990779; ***Casearia spinescens*** (Sw.) Griseb., SAL018, DB 44720, Cuba: Holguin, *S. Fuentes et al. 445* (HAJB, B), HG997955, HG997875, HG998061; ***Casearia stipitata*** Mast., SAL128, DB33096, Cameroon, *A J. M. Leeuwenberg 9884* (B), HG997994, HG997913, HG998096, FR990820; ***Caseria suaveolens*** (Poepp.) T. Samar. & M.H. Alford, SAL 153, Peru, *M. Rimachi Y. 7666* (BR), HG998005, HG997923, FR990830; ***Casearia sylvestris*** Sw., SAL004, DB 44706, Cuba, Holguin, *T. Borsch et al. 4222* (HAJB, B), HG997944; ***Casearia sylvestris*** Sw., SAL016, DB 44718, Cuba: Holguin, *SF 441* (HAJB, B), HG997953, HG997873, HG998059, FR990788; ***Casearia sylvestris*** Sw., SAL022, DB 44724, Cuba: Pinar del Rio, *T. Borsch et al. 5784* (HAJB, B), FR990792; ***Casearia sylvestris*** Sw., SAL050, DB27882, Venezuela: Guerico, *H. & E. Walter 236* (B), HG997970, HG998075; ***Casearia sylvestris*** Sw., SAL095, Colombia: La Guajira, *M.C. Martinez-Habibe et al. 2569* (UNO), HG997982, HG997902, FR990814; ***Casearia sylvestris*** Sw., SAL097, Colombia: La Guajira, *M.C. Martinez-Habibe et al. 2581* (UNO), HG997984, HG997904, HG998087, FR990815; ***Casearia sylvestris*** Sw., SAL184, Colombia: Magdalena, *M.C. Martinez-Habibe et al. 2981* (UNO), HG998014, HG997931, HG998114; ***Casearia sylvestris*** Sw., SAL188, Colombia: Magdalena, *M.C. Martinez-Habibe et al. 2987* (UNO), HG998017, HG998117, FR990837; ***Casearia sylvestris*** Sw., SAL203, Colombia: Bolivar, *M.C. Martinez-Habibe et al. 3005* (UNO), HG998020, HG997936, HG998120; ***Casearia sylvestris*** Sw., SAL208, Colombia, La Guajira, *M.C. Martinez-Habibe et al. 2733* (UNO), DB 44841; ***Casearia sylvestris*** Sw., SAL211, Colombia: La Guajira, *M.C. Martinez-Habibe et al. 2764* (UNO), HG998023, HG997939, HG998123; ***Casearia sylvestris*** Sw., SAL249, Venezuela, F. *Michelangeli & M. Alford 625* (BH); ***Casearia sylvestris*** subsp. ***myricoides*** (Griseb.) J.E. Gut., SAL010, DB 44712, Cuba: Guantanamo, *T. Borsch et al. 4901* (HAJB, B), HG997948, FR990783; ***Casearia sylvestris*** subsp. ***myricoides*** (Griseb.) J.E. Gut., SAL138, DB 44779, Cuba: Sancti Spiritu, *S. Fuentes et al. 1727* (HAJB, B), HG997998, HG997917, HG998099, FR990824; ***Casearia sylvestris*** subsp. ***myricoides*** (Griseb.) J.E. Gut., SAL245, DB 44712, Cuba, *T. Borsch et al. 5928* (HAJB, B); ***Casearia ternstroemiodes*** (Griseb.) T. Samar. & M.H. Alford, SAL 135, DB 44776, Cuba: Guantanamo, *T. Borsch et al. 4425* (HAJB, B), HG997995, HG997914, FR990821; ***Casearia tremula*** (Griseb.) Griseb. ex C. Wright, SAL 170, DB, Nicaragua: Boaco, *J. B. Quezada 242* (P), HG998008, HG997927, HG998108, FR990835; ***Casearia tremula*** (Griseb.) Griseb. ex C. Wright, SAL 213, DB 44846, Colombia: Bolivar, *V. Londono 408* (JBGP), HG998024, HG997940, HG998124, FR990842; ***Casearia ulmifolia*** Vahl ex Vent., SAL071, DB27903, French Guyana*, S. Mori 21177* (B), HG997976, HG997895, HG998079, FR990807; ***Casearia velutina*** MN078141^1^ (complete genome); ***Casearia zizyphoides*** Kunth, SAL072, DB27904, Venezuela: Bolivar, *J. A. Steyermark 88293* (B), HG997977, HG997896, HG998080, FR990808
